# A mixed-methods evaluation of a state-wide outreach perinatal mental health service

**DOI:** 10.1186/s12884-022-05229-2

**Published:** 2023-01-27

**Authors:** Sara Cibralic, Tracey Fay-Stammbach, Debbie Tucker, Deborah Song, Valsamma Eapen

**Affiliations:** 1 Ingham Institute for Applied Medical Research, Sara Cibralic, 1 Campbell St, Liverpool, NSW 2170 Australia; 2 New South Wales Ministry of Health, St Leonards, NSW Australia; 3 Western Sydney Local Health District, North Parramatta, NSW Australia; 4 University of New South Wales, Sydney, NSW Australia; 5 Central Coast Local Health District, Gosford, NSW Australia; 6 Academic Unit of Infant Child and Adolescent Services (AUCS), South Western Sydney Local Health District, Liverpool, NSW Australia

**Keywords:** Perinatal, Pregnancy, Postpartum, Treatment, Telehealth, Psychiatry

## Abstract

**Background:**

Access to perinatal mental health services in rural and remote areas is scarce, particularly perinatal psychiatry services. Telehealth, together with psychiatry consultation-liaison services are one way to improve access to areas of need. The New South Wales State-wide Outreach Perinatal Services – Mental Health (SwOPS) program is a Sydney-based program, offering specialist perinatal consultation-liaison services to rural and remote community mental health clinicians caring for perinatal women with significant mental health problems. This study aimed to evaluate healthcare practitioners’ perceptions of the SwOPS program.

**Method:**

Healthcare practitioners (*N* = 31) were purposely recruited to participate in the study. Data were analysed using a mixed-methods cross-sectional design.

**Results:**

Most participants reported being familiar with and satisfied with the service. As a result of accessing the service, participants reported an increase in knowledge and confidence regarding caring for women with moderate-to-severe or complex mental health conditions. Qualitative comments highlight the participant’s perceptions of the program.

**Conclusion:**

This study provides useful insights about a state-wide telehealth psychiatry consultation-liaison service from the perspective of practitioners. It highlights the benefits, facilitators, and barriers associated with implementing such services.

**Supplementary Information:**

The online version contains supplementary material available at 10.1186/s12884-022-05229-2.

## Introduction

Maternal mental health conditions are prevalent during the perinatal period (pregnancy and the first postnatal year), affecting approximately 20% of women [1], and are associated with a range of immediate and long term negative outcomes for women, children, and families [2–5]. The societal impacts of mental health during the perinatal period are also significant, with research showing that in Australia, the economic costs of maternal and paternal perinatal depression and anxiety alone were approximately half a billion dollars in 2012 [6], and the cost associated with not treating perinatal mental illness was close to a billion dollars in 2019 [7].

Research suggests that most of the cost associated with perinatal mental health can be attributed to the ongoing health and social impacts of the offspring rather than the parent [8] as a result of the conferred risk from parent to child [9, 10]. Consequently, Australian state (e.g., Brighter Beginnings Initiative [11]) and federal government initiatives (The National Children’s Mental Health and Wellbeing Strategy [12, 13]) have emphasised that supporting child mental health should begin with reducing risk and challenges during critical developmental periods, and as early as possible in the perinatal period. The perinatal period is a key developmental stage of illness onset (e.g. high prevalence of mental illness, parent-infant attachment problems start) that is an opportunity to engage women in mental health services and improve outcomes for both the women and her baby [12, 13].

In Australia, the most recent Mental Health Care in the Perinatal Period Australian Clinical Practice Guidelines [14] recommend that as part of standard perinatal care, all women are screened for depression and anxiety twice during pregnancy and once postnatally and that appropriate follow-up is provided for those screening high on depression and anxiety symptoms. Evidence suggests that these guidelines have been followed by many Australian perinatal healthcare providers [15–17], with a recent study showing that approximately 79% of women were screened for mental health issues during pregnancy and postnatally [17]. In remote and rural settings, however, where there are shortages in services, accessing perinatal mental health assessment and treatment services can prove to be challenging [18, 19]. This is of particular concern given research showing that women in rural and remote communities [20, 21], especially women from First Nations populations [22, 23], are more likely to experience perinatal mental health difficulties. A systematic review of 19 studies, for example, found that the prevalence of postpartum depression among rural women was around 27% [20].

During the perinatal period, women identified with moderate-to-severe mental health issues are often referred to psychiatric services [24]. Due to a limited number of suitably qualified and experienced health professionals in rural settings, some countries have utilised a psychiatry consultation-liaison service [19, 25]. Psychiatry consultation-liaison services assess women and provide feedback regarding diagnosis and management plans to the referring team who then manages the referred woman’s care [26]. In some rural and remote communities’ psychiatry, consultation-liaison services are, however, not available [19, 25]. Consequently, some countries have trailed the use of telehealth to provide psychiatry consultation-liaison service to rural and remote community health care providers [19, 25].

Telehealth refers to the use of telecommunication technologies, including telephone, video-conferencing, and internet-based applications, to provide healthcare services irrespective of location [27]. Research has shown telehealth assessment and interventions to be as effective as face-to-face interventions for rural and remote communities [28]. Minimal research has however looked at perinatal rural and remote service providers’ perspectives of psychiatry consultation-liaison services [19, 25], with only one published study being conducted on the topic [19]. In this study, Wichman, Laszewski [19] evaluated health providers’ experiences with The Periscope Project, a Wisconsin-based program offering real-time provider-to-provider teleconsultation, community resource information, and provider education. Their sample comprised 485 providers, including physicians, midwives, and nurse practitioners enrolled in The Periscope Project during the first 18 months of operation. Results showed that for the 69% of respondents that completed questionnaires on service utilisation, 100% agreed or strongly agreed that they were satisfied with their encounters with the program, the program helped them manage their patient’s care, and that the information they learned would be incorporated into the future care of patients. Given that only one published study has examined rural and remote health practitioners’ perceptions of receiving perinatal psychiatry consultation-liaison services via telehealth, more research in this area is needed.

The current study aimed to explore perinatal health care practitioners’ perceptions of the State-wide Outreach Perinatal Services – Mental Health (SwOPS) program – a telehealth psychiatry consultation-liaison service- implemented by the New South Wales state government in Australia.

## Method

### Setting

SwOPS, which is part of a broader state-wide perinatal and infant mental health service, provides free specialist consultation-liaison perinatal psychiatry services to support primarily rural and remote community mental health clinicians caring for perinatal women (and families) with moderate-to-severe or complex mental health disorders. SwOPS accepts referrals from health practitioners who have perinatal clients (pregnant or two-year postnatal) registered within their local New South Wales Health service. The most common mental health conditions that women present with are Major Depressive Disorder, Borderline Personality Disorder, Bipolar Disorder, and Generalised Anxiety Disorder.

The consultation liaison model includes a telepsychiatry session, in which the rural clinician, the perinatal woman and the SwOPS team are present. This may comprise a consultation across two, or three sites (e.g., woman’s home, community health centre and SwOPS clinic). Depending upon the referral, the consultation session may include preconception counselling, psychiatric assessment, diagnostic clarification, medication review, or care planning for mother and infant.

The service also provides clinician-to-clinician advice and support. SwOPS also offers individual clinical supervision to perinatal clinicians as well as group-based professional education in perinatal and infant mental health. These activities are aimed to build the workforce capacity of rural and remote clinicians particularly in areas which limited access to specialist perinatal psychiatry. Together, these services provided by SwOPS are aimed to improve health access and equity, and outcomes for women and their infants. In 2021 the service catered to 114 clients, the average age of women accessing the service was approximately 27 years, 14% identified as Aboriginal or Torres Strait Islander, and 2.6% were from culturally and linguistically diverse backgrounds.

### Design and Procedure

This mixed-methods cross-sectional study was approved by New South Wales Population and Health Services Research Human Research Ethics Committee (project number: 2022/ETH00284). A mixed-methods design was chosen to provide information on participants’ subjective experiences with SwOPS. Participants were recruited using purposive sampling. New South Wales Health staff were emailed a link to an online survey developed by the authors on May 13th, 2022. The survey remained open for 7 weeks, closing on June 30th, 2022.

### Measures

#### Demographic form

The demographic form included the following information: qualification, years of experience, gender, ethnicity, local health district or network of employment, classification of local health district (i.e., metropolitan, rural, or remote), and work setting (e.g., adult mental health, perinatal and infant mental health).

#### Survey

The survey contained 22 multiple-choice questions and five open-ended questions and took approximately 30 minutes to complete (see Additional file 1: Appendix 1 for the survey). The study authors prepared the survey questions based on the conceptual model of SwOPS as well as their clinical and research experience. Questions were aimed at identifying service awareness and uptake; experience with clinician-to-clinician telephone consultations; access to tertiary perinatal and infant mental health services; clinician confidence in caring for women with moderate-severe and/or complex mental health in the perinatal period; awareness of perinatal and infant mental health assessment and intervention; awareness and concern regarding mother-infant attachment; education and workforce training initiatives; liaison and supervision; and service visibility.

### Analysis

#### Quantitative analysis

Survey responses were summarised using descriptive statistics to describe participants’ perceptions of, and experiences with, SwOPS. The analysis was performed using IMB SPSS Statistics [29], version 26 for windows.

#### Qualitative analysis

Qualitative responses were analysed using an inductive thematic analysis approach drawing on an essentialist realist theoretical framework [30]. This meant that data were analysed on a semantic level rather than inferences about meaning beyond what was said by participants [30]. Two research team members (SC and DS) conducted the analysis. First, data was coded line-by-line by both authors to identify emerging themes and reach a consensus on annotations. Second, annotations were sorted into themes. Third, SC recoded all data based on the identified themes and extracted example quotes for each theme.

## Results

### Sample characteristics

Thirty-one responses to the survey were received; however, without certainty regarding the total number of service providers who refer to SwOPS, it was not possible to calculate a response rate. Of the 31 participants, 27 (87.1%) were female, and 4 (12.9%) were male. Fifteen (48.4%) participants were nurses, 14 (45.2%) were allied health professionals, and 2 (15%) were doctors. Table 1 provides information on years of professional experience, local health district classification, and work setting.

**Table 1:** Demographics

Characteristics (*N* = 31)	*n*	*%*
Years of professional experience		
1–5 years	3	9.7
5–10 years	5	16.1
10–20 years	7	22.6
20–30 years	12	38.7
30+ years	4	12.9
Local Health District classification		
Metropolitan	10	32.3
Rural	19	61.3
Remote	2	6.5
Work Setting		
Adult Mental Health -Community	9	29
Adult Mental Health -Inpatient unit	2	6.5
Child and Adolescent Mental Health	2	6.5
Drug and Alcohol	2	6.5
Child and Family Health	1	3.2
Eating Disorders	1	3.2
Perinatal and Infant Mental Health	13	41.9
Counselling	1	3.2

### Quantitative results

#### Familiarity with, and accessing, the service

Of the 31 participants, 87.1% indicated that they were somewhat familiar (48.4%, *n* = 15) or very familiar (38.7, *n* = 12) with the service, with telepsychiatry assessment and treatment planning being the most known (80.6%, *n* = 25) and most accessed service (61.3%, *n* = 19). The majority of respondents indicated that they were satisfied (16.1%, *n* = 5) or very satisfied (61.3%, *n* = 19) with the SwOPS referral process. Furthermore, most participants indicated that their clients were accepting (29%, *n* = 9) or very accepting (45.2%, *n* = 14) of the referrals to SwOPS.

#### Satisfaction with service

Most participants were satisfied (12.9%, *n* = 4) or very satisfied (64.5%, *n* = 20) with the communication from the SwOPS team, indicating that the communication was timely and very professional. With regard to treatment recommendations, 41.9% (*n* = 13) of participants indicated that they always implemented care plans and found the treatment recommendations helpful. Most participants also indicated that clients were accepting (29%, *n* = 9) or very accepting (35.5%, *n* = 11) of accessing the service via telehealth and that clients were always (41.9%, *n* = 13) or often (25.8%. *n* = 8) comfortable with the service. Furthermore, 83.9% (*n* = 26) of participants indicated that they would recommend SwOPS to colleagues.

More than half of the participants (58.1%, *n* = 18) indicated that they had accessed SwOPS clinician-to-clinician telephone consultation service, and all found it beneficial (*n* = 3) or very beneficial (*n* = 15). Twenty-nine percent of participants (*n* = 9) indicated that they had accessed SwOPs education services, and the majority (*n* = 7) indicated their learning needs met. Approximately 61% (*n* = 19) of participants accessed SwOPS consultation liaison support, with most (*n* = 18) indicating that they found the service effective. Furthermore, 54.8% (*n* = 17) of participants noted that they had accessed SwOPS regarding mother-infant attachment concerns, with most (*n* = 14) indicating that the SwOPS team had addressed the concerns and that the consultation led to an increased understanding of the mother-infant attachment relationship (*n* = 16).

#### Confidence and knowledge

The majority of participants (80.6%, *n* = 25) indicated that the feedback provided by SwOPS increased their confidence in caring for women with moderate-to-severe or complex mental health difficulties during the perinatal period. Furthermore, most participants indicated that their knowledge regarding perinatal and infant mental health assessment and treatment had increased (48.4%, *n* = 15) or significantly increased (12.9, *n* = 4), as had their knowledge regarding the infant-mother attachment relationship (51.9%, *n* = 16), after accessing SwOPS.

#### Barriers to accessing SwOPS

Seven participants (22.6%) indicated that they experienced barriers with regard to accessing SwOPS. The types of barriers encountered we elaborated on in the qualitative responses.

### Qualitative results

Five themes were identified from the qualitative response: “Valuable service”, “wider range of education services”, “feedback and confidence”, “barriers to accessing SwOPS”, and “service improvements”.

#### Valuable service

Consistent with quantitative data indicating that most participants were satisfied with the services, most participants qualitative responses indicated positive perceptions about the service:

*“I cannot speak highly enough of the SwOPS service! The team is absolutely wonderful and a God-send to our clients, who also speak very highly of their appointments. The team hold our clients in high, positive regard, and their warmth and understanding and non-judgemental approach is so very appreciated by our new mums.” (Participant 28, allied health professional, female)*

*“This is a wonderful service for clinicians in rural and remote areas to access professional support with a highly vulnerable group.” (Participant 6, allied health professional, female)*

*“I have found the SwOPS service highly professional with an excellent ability to quickly engage with clients via telehealth.” (Participant 1, nurse, female)*

#### Wider range of education topics

SwOPS providing a wider range of education topics was another theme that emerged from qualitative responses. Participants’ responses indicated that they wanted more education from SwOPS on topics such as the parent-child attachment relationship, “medication”, “intervention” and “treatment” planning, “other mental health conditions” as well as more information regarding the SwOPS service (e.g., referral pathways, services provided).

*“Anything is great, but perhaps something [regarding] other mental health conditions (e.g., Borderline Personality Disorder) and their interaction with Postnatal Depression.” (Participant 28, allied health professional, female)*

*“Parent/infant dyad, the mental health of the dyad. Difficult cases requiring mental health involvement, admission and management throughout the pregnancy” (Participant 30, allied health professional, female)*

#### Feedback and confidence

Qualitative responses revealed that for participants where SwOPS feedback did increase confidence, the main reasons were that the recommendations were provided by “experts”, they were “appropriate”, and they helped with service planning. Furthermore, participants indicated that SwOPS recommendations helped increase engagement of other services (e.g., Mental Health Services) in the care of their clients. Only one participant provided a justification for indicating that SwOPS did not increase her confidence, noting that she was an experienced clinician who accessed SwOPS to validate “what [she] is already doing”.

*“I have learned from sitting in on consultations and been able to apply that learning to individual clients and incorporate into practice with other women with similar diagnosis … Women also feel more confident having heard advice from an expert team making it easier to implement those expert recommendations” (Participant 19, nurse, female)*

*“[SwOPS] helped to keep … mental health services engaged … [and] to see that pregnancy and [the] postnatal period [are] a time of risk of relapse that requires planning and support rather than discharging … ” (Participant 30, allied health professional, female)*

#### Barriers to accessing SwOPS

While quantitative responses indicated that barriers were experienced by several participants trying to access SwOPS services, qualitative responses revealed that these barriers included poor awareness of service and referral pathways and that engaging with the service was time-consuming.

*“Lack of awareness of the service and integration of referral pathway into clinical practice” (Participant 14, nurse, make)*

#### Service improvements

Fourteen participants provided recommendations for service improvements, with most participants indicating that increasing awareness of the service would help improve the service:

*“More marketing and promotion about the service” (Participant 16, nurse, female)*

*“I understand that we have been in a global pandemic and the restrictions this has placed … this has meant that the service is not widely known about nor what is on offer. Could a traveling road show be the way to increase the profile.” (Participant 5, nurse, female)*

One participant also noted that it would be of benefit to her client population if SwOPS were to have an Aboriginal Health Worker:

*“Coming from an area with a high First Nations population, I feel it would be appropriate for SwOPS to have an Aboriginal Health Worker who could participate to help patients to feel culturally safe. Often telehealth can compound feelings of not feeling culturally safe and the service isn't taken up. If I was able to tell women that ‘Aunty’ was also going to be present I think they would be much more comfortable using it.” (Participant 29, allied health professional, female)*

## Discussion

This study aimed to evaluate the SwOPS program. Study results provide descriptive findings on health practitioners’ experiences with, and perspectives of, SwOPS. The findings indicate that the majority of participants were familiar and satisfied with the program and had accessed a range of services offered by the program (e.g., telepsychiatry assessment and treatment service, clinician-to-clinician consultation service, and consultation-liaison support service, and education). It was also found that accessing the service increased practitioner knowledge and confidence in caring for women with moderate-to-severe mental or complex health conditions as well as expertise regarding the infant-mother attachment relationship, a factor that has been shown to play a protective role in the overall health and wellbeing of children [31, 32]. The qualitative findings suggest that although most participants held the service in high regard, there are ways that the service could be improved, including increasing awareness of the service as well as having an Aboriginal Health Worker as part of the SwOPS team who could assist in supporting women from First Nations populations.

These findings highlight the important role that SwOPS plays in enhancing services to rural and remote regions of New South Wales, Australia. Given the shortage of services in rural and remote areas [18, 19] and the greater risk of perinatal mental health difficulties observed in these populations [20, 21], it was of no surprise that the most accessed service was the telepsychiatry assessment and treatment service. This finding indicates that the implementation of telepsychiatry can help provide assessment and treatment services to women who otherwise would not have access to such specialty care. The finding is consistent with previous research showing that service providers utilise and are satisfied with telepsychiatry services [19]. In addition, the finding that practioner knowledge and confidence in caring for women with moderate-to-severe or complex mental health difficulties increased indicates that programs such as SwOPS can play a role in upskilling local health professionals in caring for women during the perinatal period. This again is of particular importance given the shortage of specialist services available in rural and remote areas.

Though, not a major theme to emerge from the data, it was interesting that one participant suggested that an Aboriginal Health Worker may increase First Nations women’s utilisation of services such as SwOPS. Past research has found that First Nations women are at a greater risk of experiencing perinatal mental health difficulties [22, 23]. One participant in this study felt that having an Aboriginal Health Worker could help First Nations women feel culturally safe when accessing the service. Due to a history of trauma experienced by First Nations populations, including massacres, being removed from traditional lands, and the forced removal of children (known as the Stolen Generation) [33], it is understandable why some First Nations women in Australia would not feel comfortable accessing government-run health services. Past research has found that Aboriginal Health Workers can positively affect communication between health professionals and patients and increase access to culturally safe care, which can help address the health inequalities experienced by First Nations populations [34].

### Strengths and limitations

Strengths of this study include the use of a mixed-methods methodology and that the study is, to our knowledge, the second study to have evaluated a telehealth perinatal psychiatry consultation-liaison service. Using a mixed-methods methodology allowed researchers to identify the extent, as well as provide a more in-depth understanding (through the use of qualitative questions), of service utilisation and perceptions regarding service. This study also had a number of limitations that must be acknowledged. First, as the study is descriptive, inferential conclusions cannot be drawn from the data. Second, the small number of participants reduces the generalisability of findings. Third, also negatively impacting the generalisability of findings, was that only a small number of participants (*n* = 2) worked within remote areas. Fourth, as there is an unequal distribution of health practitioners that responded to the survey, with the majority of respondents being allied health professionals and nurses, the results may not be representative of all health professionals supporting perinatal women in rural and remote settings. Fifth, this study only explored the perspectives of health practitioners. The perspectives of women being referred to the service would be particularly useful in evaluating and improving the service.

## Conclusion

Taken together, results of this study provide insight into the benefits, facilitators, and barriers associated with implementing a statewide telehealth perinatal psychiatry consultation-liaison service, as percieved by allied health professionals and nurses accessing the service. More research with larger sample sizes would help increase the generalisability of finidings. Further, research undertaken with perinatal women referred to the services would help with further service evaluation and improvement.

## Supplementary Information

**Additional file 1.**

## Data Availability

Data from the current study are available from the corresponding author on reasonable request.

## Abbreviation

SwOPS: State-wide Outreach Perinatal Services – Mental Health
