# Supplementary material for: A mixed-methods evaluation of a state-wide outreach perinatal mental health service
Source: BMC Pregnancy Childbirth. 2023 Jan 27;23:74. doi: 10.1186/s12884-022-05229-2 (PMC9881293; doi:10.1186/s12884-022-05229-2)
Supplement: Supplementary file 1 — Additional file 1. [file 12884_2022_5229_MOESM1_ESM.docx]

**Appendix 1**


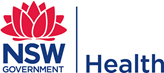


SwOPS review 2022

**Survey Questions**

# Demographic Questions

- 1. I am a:
- Doctor (please specify specialty: ______ )
- Nurse (please specify specialty: ______ )
- Allied health professional (please specify: _______)
  1. Years of professional experience:
- < 1
- 1-5
- 5-10
- 10-12
- 20-30
- 30 +
  1. Your gender:
- Male
- Female
- Other (please specify)
  1. Your ethnicity: _______________
  2. The local health district or network you work in:
- [Central Coast](https://www.health.nsw.gov.au/lhd/pages/cclhd.aspx)
- [Illawarra Shoalhaven](https://www.health.nsw.gov.au/lhd/pages/islhd.aspx)
- [Nepean Blue Mountains](https://www.health.nsw.gov.au/lhd/pages/nbmlhd.aspx)
- [Northern Sydney](https://www.health.nsw.gov.au/lhd/pages/nslhd.aspx)
- [South Eastern Sydney](https://www.health.nsw.gov.au/lhd/pages/seslhd.aspx)
- [South Western Sydney](https://www.health.nsw.gov.au/lhd/pages/swslhd.aspx)
- [Sydney](https://www.health.nsw.gov.au/lhd/pages/sydlhd.aspx)
- [Western Sydney](https://www.health.nsw.gov.au/lhd/pages/wslhd.aspx)
- [Far West](https://www.health.nsw.gov.au/lhd/pages/fwlhd.aspx)
- [Hunter New England](https://www.health.nsw.gov.au/lhd/pages/hnelhd.aspx)
- [Mid North Coast](https://www.health.nsw.gov.au/lhd/pages/mnclhd.aspx)
- [Murrumbidgee](https://www.health.nsw.gov.au/lhd/pages/mlhd.aspx)
- [Northern New South W](https://www.health.nsw.gov.au/lhd/pages/nnswlhd.aspx)ales
- [Southern New South W](https://www.health.nsw.gov.au/lhd/pages/snswlhd.aspx)ales
- [Western New South W](https://www.health.nsw.gov.au/lhd/pages/wnswlhd.aspx)ales
- Justice Health and Forensic Mental Health Network
  1. Your LHD is classified as:
- Metro
- Rural
- Remote
  1. Your work setting is:
- Adult MH Community
- Adult MH Inpatient units
- Psychiatry (e.g., Psychiatry CL)
- CAMHS
- Drug and Alcohol
- Substance Use in Pregnancy and Parenting
- Child and Family Health
- Sustained Home Nurse Visiting
- Aboriginal Maternal Infant Health
- Eating Disorders
- Perinatal and Infant MH
- Maternity
- Neonatal Care
- General Practitioner
- Other (please specify): ________________________

**Evaluation Questions**

***Service awareness and uptake***

1. How familiar are you with the SwOPS service and what they offer?

Very familiar with the service

Somewhat familiar

Not at all familiar

1. If familiar, where did you first hear about the service?
2. SwOPS offers several services; please indicate which service/s you are aware of? (Tick all that apply)

Pre-conception counselling

Staff education and training

Support for care co-ordination and case conferencing

Assessment and support for mother-infant attachment

Tele-psychiatry assessment and treatment planning

Telephone consultations for clinician support and liaison

Mental health birth planning

1. Which SwOPS services have you sought and used most? (Tick all that apply)

Pre-conception counselling

Staff education and training

Support for care co-ordination and case conferencing

Assessment and support for mother-infant attachment

Tele-psychiatry assessment and treatment planning

Telephone consultations for clinician support and liaison

Mental health birth planning

1. How many clients have you referred to SwOPS?

1 client

2- 5 clients

5- 10 clients

10+ clients

None

1. Are there any barriers to accessing SwOPS services?

Yes - please describe

No

***Clinician to clinician telephone consultation***

1. Have you contacted the SwOPS team for advice via telephone

Yes

No

If yes, how many times have you contact the SwOPS team via phone

1

2-5

5-10

10+

How beneficial have you found the SwOPS phone consultations:

Very beneficial

Beneficial

Neutral

Unbeneficial

Very unbeneficial

***Access to tertiary PIMH services***

*Please answer the following questions based on your experience of using SwOPS generally or across multiple contacts with SwOPS. If you have only used SwOPS once, then base your answers on that single contact.*

1. Overall, how satisfactory has your experience with making a SwOPS referral been:

Very satisfactory

Satisfactory

Neutral

Unsatisfactory

Very unsatisfactory

1. What is your impression of using the SwOPs tele-health equipment?

Very easy to navigate

Somewhat easy to navigate

Neutral

Somewhat difficult to navigate

Very difficult to navigate

Overall, how accepting have clients been of SwOPS referrals?

Very accepting

Accepting

Neutral

Unaccepting

Very unaccepting

Overall, how accepting have clients been of receiving psychiatric services via tele-health?

Very accepting

Accepting

Neutral

Unaccepting

Very unaccepting

1. Overall, how satisfactory has the communication from SwOPS been (e.g., appointment info, advice, reports):

Very satisfactory

Satisfactory

Neutral

Unsatisfactory

Very unsatisfactory

1. Overall, how timely has the communication (e.g., appointment info, advice, reports) from SwOPS been:

Very timely

Timely

Neutral

Untimely

Very untimely

1. Overall, how professional has the communication from SwOPS been?

Very professional

Professional

Neutral

Unprofessional

Very unprofessional

1. How long did you have to wait for a consultation after you referred to the service?

1-2 weeks

2-4 weeks

1-2 months

2-4 months

1. Did you receive consultation follow-up or a review after the initial consultation?

Yes

No

N/A

1. If yes, how satisfied were you with the consultation follow-up?

Very satisfactory

Satisfactory

Neutral

Unsatisfactory

Very unsatisfactory

1. Overall, how frequently have you implemented the care plan and/or advice provided by SwOPS?

Always

Often

Sometimes

Never

1. Overall, were the treatment recommendations helpful for your client, and improved the client outcome?

Always

Often

Sometimes

Never

Unsure

1. Overall, was the client comfortable with the SwOPS consultation and recommendations?

Always

Often

Sometimes

Never

Unsure

1. Would you recommend SwOPS to a colleague?

Yes

No – why not?

***Confidence in caring for women with mod-severe and/or complex mental health in the perinatal period***

1. Did the feedback/advice provided by the SwOPS team assist you to feel more confident in supporting the client

Yes, how so?

No, why not?

***Awareness of perinatal and infant mental health assessment and intervention***

1. After using SwOPS my knowledge about perinatal and infant mental health assessment and treatment compared to earlier, was:

Increased significantly

Increased

Unchanged

Decreased

Significantly decreased

1. After using SwOPS my confidence in caring for women and infants in the perinatal period compared to earlier was

Much higher

Higher

Unchanged

Lower

Much lower

***Mother-infant attachment***

Were you concerned about the quality of the mother-infant attachment relationship (e.g., disrupted attachment) of clients you referred:

Yes

No

1. If yes, did the SwOPS feedback address these concerns?

Yes

No

1. Has your engagement with SwOPS enhanced your understanding of mother-infant attachment?

Definitely yes

Probably yes

Unsure

Probably no

Definitely no

***Education and workforce training initiatives***

1. Have you participated in any education offered by SwOPS?

Yes

No

1. If yes, Did the education meet your learning needs or expectations?

Yes

No, why not?

1. What education from SwOPS would you find useful?
2. What delivery mode would work best i.e.: face to face, PEXIP, Teams meeting?

***Liaison and supervision***

1. Did SwOPS provide consultation liaison support?

Yes

No

1. If so, was the consultation liaison effective?

Definitely yes

Probably yes

Unsure

Probably no

Definitely no

***Visibility***

1. How known is SwOPS to other professionals or services?
   - Not at all known to other professionals or services
   - Only minimally known to other professionals or services
   - Highly known to other professionals or services
2. What will assist SwOPS becoming more widely known and utilised?

- More marketing and promotion about the service e.g., flyers, emails, road show
- Change of name to make it more known or recognisable e.g.: use of Perinatal and Infant Mental Health in the name?
- Perinatal and infant mental health clinicians should raise its profile more
- Other, please specify:____________________

1. What are some recommendations for service improvement?
2. Do you have any other comments about SwOPS you would like to share?
